# Knee joint position sense and kinematic control in relation to motor competency in 13 to 14-year-old adolescents

**DOI:** 10.1186/s13052-024-01765-z

**Published:** 2024-09-29

**Authors:** Yan-Ci Liu, Patrick Esser, Benjamin David Weedon, Daniella Springett, Shawn Joshi, Meng-Hsuan Tsou, Ray-Yau Wang, Helen Dawes

**Affiliations:** 1 School and Graduate Institute of Physical Therapy, College of Medicine, National Taiwan University, No.17, Xuzhou Rd., Zhongzheng Dist., Taipei, 100 Taiwan; 2 Centre for Movement, Occupation and Rehabilitation Sciences, Oxford Brookes University, Oxford, UK; 3 Oxford Institute of Nursing, Midwifery and Allied Health Research (OxINMAHR), Oxford Brookes University, Oxford, UK; 4 School of Biomedical Engineering, Science and Health Systems, Drexel University, Philadelphia, PA USA; 5 Department of Physical Therapy and Assistive Technology, National Yang Ming Chiao Tung University, Taipei, Taiwan; 6 Medical School, NIHR Exeter BRC, University of Exeter, Exeter, UK; 7 Nuffield Dept of Clinical Neurosciences, University of Oxford, Oxford, UK; 8 Physical Therapy Center, National Taiwan University Hospital, Taipei, Taiwan

**Keywords:** Joint position sense, Proprioception, Motor control, Developmental coordination disorder, Motor competence

## Abstract

**Background:**

Motor competence (MC) is a key component reflecting one’s ability to execute motor tasks and is an important predictor of physical fitness. For adolescents, understanding the factors affecting MC is pertinent to their development of more sophisticated sporting skills. Previous studies considered the influence of poor proprioceptive ability on MC, however, the relationship between lower limb joint position sense, kinematic control, and MC is not well understood. Therefore, the aim of this study was to determine the relation between joint position sense and kinematic control with MC in adolescents during a lower limb movement reproduction task.

**Methods:**

This study was a cross-sectional design. Young people (*n* = 427, 196 girls and 231 boys) aged 13 to 14 years were recruited. A movement reproduction task was used to assess joint position sense and kinematic control, while the Movement Assessment Battery for Children (mABC-2) was used to assess MC. In this study, participants were categorized into the Typically Developed (TD, *n* = 231) and Probable Developmental Coordination Disorder (DCD, *n* = 80) groups for further analysis of joint position sense, kinematic control, and MC between groups.

**Results:**

Kinematic data, specifically normalized jerk, showed a significant correlation with MC. There was no correlation between knee joint position sense and MC, and no group differences between DCD and TD were found.

**Conclusions:**

Joint position sense should not be used as a measure to distinguish TD and DCD. Rather than joint position sense, control of kinematic movement has a greater influence on the coordination of the lower limbs in adolescents. Movement control training should be implemented in the clinical setting to target kinematic control, rather than focus on joint position sense practice, to improve motor competency.

**Trial Registration Identifier:**

NCT03150784. Registered 12 May 2017, https://clinicaltrials.gov/study/NCT03150784.

**Supplementary Information:**

The online version contains supplementary material available at 10.1186/s13052-024-01765-z.

## Background

Motor competence (MC) encompasses the proficiency to execute various motor tasks, mastery levels of both fine and gross motor skills [1]. MC is a key component in children and adolescents in developing a healthy, active lifestyle and is a predictor of physical fitness [1, 2]. Enhanced MC correlates with increased engagement in physical activity, while diminished MC aligns with decreased physical activity levels [1]. Despite extensive focus on external factors that affect MC, such as physical education class and environmental interplay, the association between MC and intrinsic movement factors remains understudied.

Proprioception, encompassing limb coordination and motor planning, refers to the intrinsic awareness of relative body limb position and motion [3], encompassing joint position sense and kinesthesia [4]. Joint position sense, tested through active or passive reproduction of limb positioning, enables individuals to identify and replicate limb position in space [4]. Crucial for maintaining dynamic joint stability and preventing injury [5], kinematic control refers to the ability to regulate gross limb movement, aiming to reduce musculoskeletal system degrees of freedom task-dependently [6, 7]. Kinematic analysis provides insight into the motor control mechanisms underlying movement [6].

Changes in joint position sense and kinematics have been linked to limb sensory control impairments during movements [8]. However, little is known about the relationship between joint position sense, kinematic control, and MC, particularly of the lower limb in adolescents. Further, for adolescents with difficulty in motor skill and sensory processing, joint position sense and kinematic control may also demonstrate impairments. Children and adolescents with developmental coordination disorder (DCD) exhibit impaired upper and lower limb joint position sense compared to typically developing (TD) peers [9, 10], alongside inferior kinematic control during movements, characterized by lower reaction times, larger errors, longer movement and/or deceleration times, curved trajectories, and greater movement speed variability [10]. While a link between decreased MC and poor joint position awareness is speculated, previous research on proprioceptive deficits in DCD remains inconclusive [11–13], with some indicating poorer joint awareness in DCD adolescents [12, 14], while others found no differences [15]. Moreover, weak correlations exist between MC (mABC-2) scores and upper limb joint position sense in both TD and DCD children [13–15]. Yet, studies mainly focused on upper extremity joint position sense in younger children, leaving a gap in understanding lower extremity performance in adolescents [16]. Understanding how MC, joint position sense, and kinematic control influence motor skill development is crucial for tailored interventions. Our study aims to assess the relations between joint position sense and kinematic control with MC, and to understand the differences in these measures in adolescents with and without DCD. We predict that lower limb joint position sense and kinematic control reflect MC levels in both TD and DCD adolescents.

## Methods

### Study design

This study was a cross-sectional design. Baseline data, strength, and power of each participant were collected at the start of the experiment. Participants were then instructed to do a movement reproduction task, and lastly completed a motor competence assessment. After collection of all data, participants were categorized into the probable DCD group and TD group, and results between the two groups were compared. The study was approved by the University Research Ethics Committee (UREC Registration No: 161033) and conducted in accordance with the Declaration of Helsinki (1964) and later revisions. The study followed the Research Governance Framework for Health and Social Care (DoH 2nd Edition, July 2005) and is registered at ClinicalTrials.gov. (Identifier: NCT03150784).

### Participants

This study focused on children aged 13–14 years, a critical age when activity levels reduce and when sporting skills are being built. Data were collected from a whole year group across three mainstream secondary schools in Oxfordshire, UK. A total of 427 participants were included, meeting the following criteria: (1) normal intelligence reported by teachers, and (2) no contraindications to maximal physical exercise identified by the Physical Activity Health Questionnaire (PARQ). Exclusion criteria included children with muscular/neurological degenerative conditions, uncontrolled epilepsy/seizures (must have stable epilepsy or be on medication for over 12 weeks), or recent surgery within six months. Parents/guardians were encouraged to consult the respective GP/paediatrician/physiotherapist if safety concerns arose regarding a child’s participation.

### Inertial measurement unit

A single inertial measurement unit (IMU) (LPMS-B2, LP-RESEARCH Inc. Tokyo, Japan) was used to detect angular movement of the knee joint. The IMU has a built-in 3-axial accelerometer, gyroscope, and magnetometer to detect three-dimensional orientation. The device was connected to a PC via Bluetooth 4.1, and data were recorded at a sampling rate of 100 Hz. Movement IMU data were analyzed using a custom program written in LabVIEW2011 (National Instruments, Ireland).

### Active movement reproduction task

**Fig. 1:**
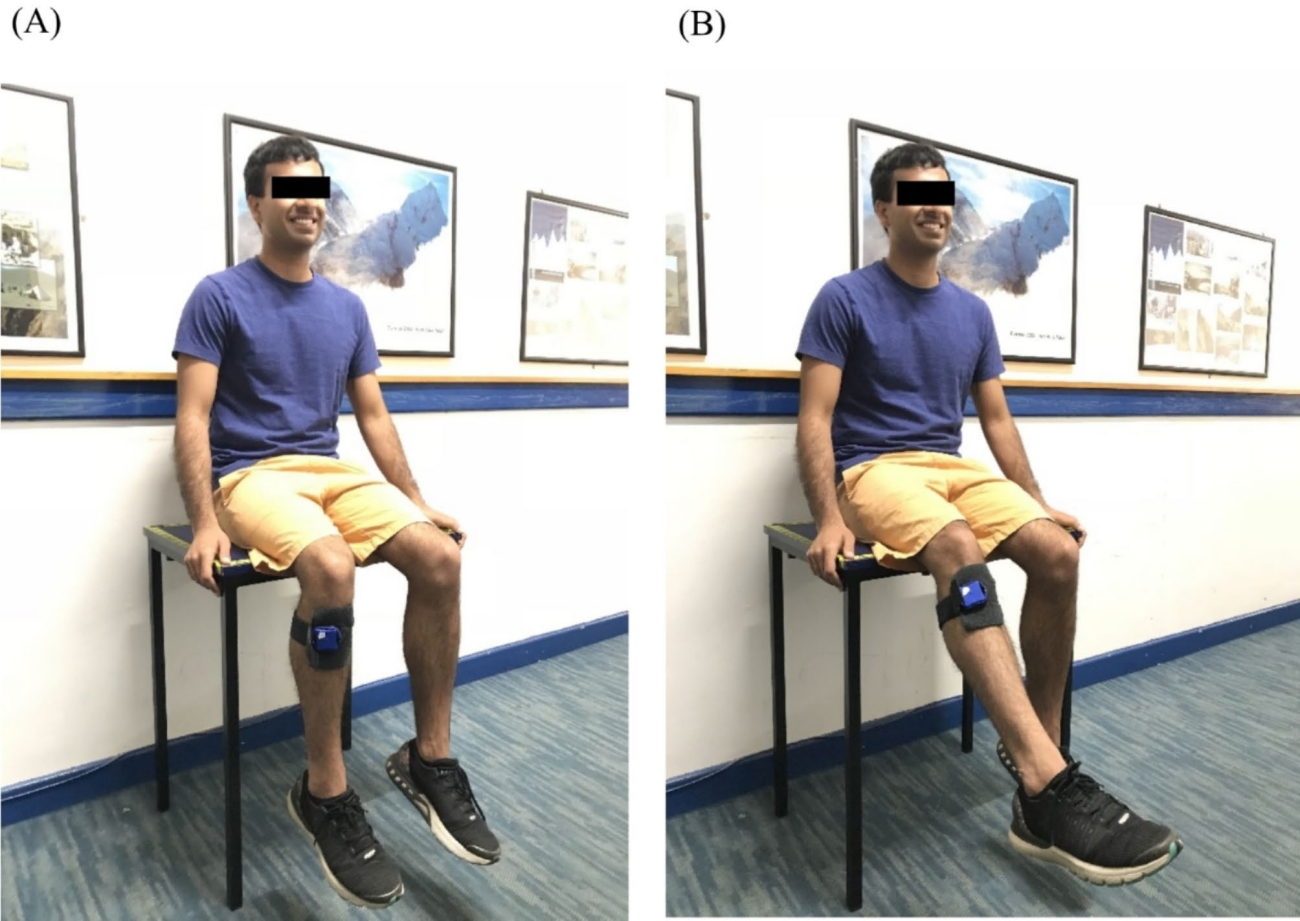
The starting position of movement reproduce task. (**A**) Participants were instructed to sit on a table with their leg relaxing and both hands holding the table for stability. (**B**) Participants repeated the target angle actively and instructed to hold the new angle for 3 s

To test for joint position sense and kinematic measures, participants were asked to execute an active movement production task. Active movement reproduction tasks are widely used in clinical practice and is the most common method of measuring joint position and movement [17]. In our study, the single IMU was attached to the participant’s tibial bone (approximately 5 cm below the patella) of their dominant leg (Fig. 1a), which was defined as the leg they would prefer to kick a football. The researcher passively moved the subject’s knee to approximately 30° of knee flexion from the 90° starting position of knee flexion and maintained this new position for 3 s. The leg was then moved back to the 90^o^ starting position of knee flexion. Participants were then asked close their eyes to eliminate any visual cues, and reproduce the knee movement actively and maintain the new angle for 3 s. Each participant repeated this twice and keeping their eyes closed during trials (Fig. 1b).

### Measures

Descriptive measures, including age, gender, height, weight, BMI, leg length, grip strength, shoe size, broad jump, and motor competence, were recorded during the baseline assessment. Strength and power were measured by grip strength and broad jump, respectively [18]. mABC-2, which included manual dexterity (MD), aiming & catching (AC), and balance (B), was used to determine motor competence [19]. Data from the IMU were used to assess joint position sense and kinematic performance. To calculate joint position sense, the absolute angle error (AE, the absolute difference between reference and reproduce angle) and standard deviation between two repeats (SDPE) during the movement reproduction task were recorded. Three-dimensional orientation was extracted from the sensor, from which angular change in the Euler X plane was derived. Movement time (MT) was derived from the minimum to maximum of angular change [20]. Angular velocity was derived and smoothened using the Savitzky-Golay filter (3rd polynomial, 2nd order), from where peak velocity (PV [deg/s] [21]) and corresponding time, expressed as a percentage of the overall movement, (PTPV) were taken. For kinematic performance, movement time (MT), peak angular velocity (PV), and percentage of peak angular velocity (%PV) were also estimated by the data extracted from the IMU. Movement smoothness was assessed by normalized angular jerk [deg/s^3] which was derived from double differentiation of angular velocity using the same Savitzky-Golay filter at each differentiation. Jerk was normalized according to formula (1), providing normalized jerk (NJ) [22–24].

1 $${\text{NJ}} = \sqrt {\left( {\frac{1}{2}{\text{*}}\mathop {\mathop {\int \: }\limits^{T\:end} }\limits_{T\:start} Jer{k^2}\left( t \right)dt} \right)*Duratio{n^5}/Lengt{h^2}}$$

### Data analysis

Descriptive statistics (mean ± standard deviation or frequency) were generated for all variables, and all statistical analysis was done using SPSS 19.0 (IBM Corp, Armonk, NY). We used Pearson correlation to determine the relation between joint position sense (AE, SDPE), motor competence (mABC-2), and kinematic control (MT, PV, %PV). The Levene’s and Kolmogorov-Smirnov tests were used to test homogeneity and normality. The Mann-Whitney U test was used to calculate the gender or motor competence group differences (adolescents who scored below the 5th percentile were determined as probable DCD [25]; those above the 25th percentile were determined as TD) of all measures. Significance level was set as *p* < 0.05 for all tests.

## Results

There were 427 adolescents (13–14 years old) who participated in this study, including 196 girls and 231 boys. Table 1 shows the descriptive data of all participants. Compared to girls, boys were taller (Cohen’s d = 0.61; *p* < 0.001), fitter (Cohen’s d = 0.15; *p* = 0.017) and had stronger performance in both grip strength (Cohen’s d = 0.47; *p* < 0.001) and broad jump (Cohen’s d = 0.49; *p* < 0.001). Boys scored higher in motor competence according to the mABC-2 in manual dexterity (Cohen’s d = 0.43; *p* = 0.004) and aiming & catching (Cohen’s d = 0.95; *p* < 0.001) categories, but there were no gender differences in either the balance category or the total score of the mABC-2 (*p* > 0.05).

**Table 1:** Descriptive data (*n* = 427)

	Total (*n* = 427)	Boys (*n* = 231)	Girls (*n* = 196)	*p* value
Gender (male/female)	231/196			
Height(cm)	162.13 ± 8.71	166.41 ± 8.56	161.44 ± 7.74 *	< 0.001
Weight (kg)	54.73 ± 12.15	55.67 ± 13.20	53.63 ± 10.71	0.206
BMI	20.31 ± 4.49	20.00 ± 4.05	20.67 ± 4.94 *	0.017
Leg length (cm)	88.22 ± 5.94	89.27 ± 6.04	86.98 ± 5.57 *	< 0.001
Grip Strength (kg )	24.28 ± 6.89	25.73 ± 7.91	22.58 ± 4.96 *	< 0.001
Broad Jump (m)	1.60 ± 0.27	1.66 ± 0.26	1.53 ± 0.27 *	< 0.001
mABC-2				
Manual dexterity	21.04 ± 6.82	24.70 ± 5.35	22.11 ± 6.63 *	0.004
Aiming & Catching	17.67 ± 4.92	19.52 ± 4.53	15.20 ± 4.34 *	< 0.001
Balance	29.22 ± 6.23	28.98 ± 5.89	29.47 ± 6.65	0.079
Total score	67.93 ± 11.94	73.20 ± 10.96	66.79 ± 12.12	0.082

Data shown as mean ± SD

*, significant gender difference

Results of knee joint position sense and kinematic performance are shown in Table 2. The mean value of AE is 5.52 ± 3.80° and SDPE is 1.46 ± 1.67. There were no gender differences in the joint position sense and kinematic data except for the movement time (Cohen’s d = 0.16; *p* = 0.022), which showed boys taking less time to reach the target angle.

**Table 2:** Joint position sense and kinematic control analysis (*n* = 427)

Variables	Total	Boys (*n* = 231)	Girls (*n* = 196)	*p* value
Joint position sense				
Absolute error (°)	5.52 ± 3.80	5.60 ± 3.92	5.44 ± 3.66	0.842
Position sense error variability (SDPE)	1.46 ± 1.67	1.38 ± 1.08	1.55 ± 2.17	0.772
Kinematic control				
Movement time (s)	1.46 ± 0.39	1.43 ± 0.40	1.49 ± 0.37*	0.022
Peak velocity (°/s)	69.45 ± 19.72	70.90 ± 120.67	67.74 ± 18.45	0.175
% of peak velocity (%)	39.82 ± 9.52	39.33 ± 9.96	40.40 ± 8.96	0.126
Normalized Jerk (m/s^3^)	9390814.86 ± 17560673.16	16732067.91 ± 238,728,000	738623.77 ± 3006903.63	0.970

Data shown as mean ± SD

*, significant difference of gender difference

Table 3 shows the correlation between joint position sense, MC, and kinematic control. AE was positively correlated with SDPE (*r* = 0.155, *p* = 0.001) but there was no correlation between knee joint position sense (AE & SDPE) and MC (mABC-2 categories score and total score) (*p* > 0.05). The kinematic data showed significant correlation with MC. MT was negatively correlated with manual dexterity (*r*=-0.102, *p* = 0.035) and total score of mABC-2 (*r*=-0.108, *p* = 0.025). PV was negatively correlated with the balance category in mABC-2 (*r*=-0.098, *P* = 0.044) and NJ was negatively correlated with manual dexterity (*r*=-0.101, *p* = 0.037), balance (*r*=-0.159, *p* = 0.001), and the total score of mABC-2 (*r*=-0.176, *p* < 0.001) (Table 2).

**Table 3:** Correlation between joint position sense, kinematic control, and motor competence

	AE	SDPE	MT	PV	%PV	NJ	mABC-2-MD	mABC-2-AC	mABC-2-B	mABC-2-total
**Joint position sense**										
AE		0.155**	-0.043	-0.179**	-0.027	0.190**	-0.015	-0.027	-0.024	-0.032
SDPE			0.191**	-0.086	0.046	0.096*	-0.058	-0.048	-0.061	-0.085
**Kinematic control**										
MT				-0.618 **	-0.288**	0.325**	-0.102*	-0.082	-0.031	-0.108*
PV					0.202**	-0.142*	0.003	0.015	-0.098*	-0.043
%PV						-0.054	0.046	-0.074	0.006	-0.001
NJ							-0.101*	-0.085	-0.159**	-0.176**

*, *p* < 0.05; **, *P* < 0.001

Abbreviations: AE, Absolute error; SDPE, Position sense error variability; MT, movement time; PV, Peak velocity; %PV, percentage time to peak velocity; NJ, Normalized jerk; MD, manual dexterity; AC, aiming & catching; B, balance

Eighty children met the specific criteria according to the mABC-2 and were assigned into the DCD group while 231 children were categorized as TD. Adolescents with DCD had higher BMI (Cohen’s d = 0.40; *p* = 0.003), shorter leg length (Cohen’s d = 0.49; *p* < 0.001), lower grip strength (Cohen’s d = 0.23; *p* = 0.038), and lower lower-limb power (Cohen’s d = 0.69; *p* < 0.001) compared to their TD counterparts (Table 4). However, there were no significant group differences in joint position sense and kinematic control (Table 5).

**Table 4:** Descriptive data of DCD and TD group

Variance	DCD group (*n* = 80)	TD group (*n* = 231)	*p* value
Height (cm)	162.86 ± 8.61	164.20 ± 7.71	0.074
Weight (kg)	56.38 ± 12.67	53.32 ± 11.57	0.071
BMI (kg/m^2^)	21.18 ± 4.03	19.70 ± 3.63	0.003
Leg length (cm)	86.04 ± 6.20	88.76 ± 5.24	< 0.001
Shoe size (UK)	6.32 ± 2.09	6.67 ± 1.93	0.088
Grip strength (kg)	22.75 ± 7.43	24.33 ± 6.64	0.038
Broad jump (m)	1.46 ± 0.26	1.64 ± 0.26	< 0.001

Data shown as mean ± SD

DCD, Developmental coordination disorder; TD, typical development

**Table 5:** Joint position sense and kinematic control analysis of DCD and TD group

Variance	DCD group (*n* = 80)	TD group (*n* = 231)	*p* value
Joint position sense			
Absolute error (°)	5.84 ± 3.36	5.56 ± 3.69	0.904
Position sense error variability (SDPE)	1.73 ± 1.89	1.35 ± 1.14	0.093
Kinematic control			
Movement time (s)	1.53 ± 0.50	1.42 ± 0.35	0.183
Peak velocity (°/s)	71.91 ± 24.22	69.24 ± 19.35	0.331
% of peak velocity (%)	39.92 ± 9.39	39.81 ± 9.25	0.879
Normalized Jerk (m/s^3^)	47231111.41 ± 405,557,000	732468.17 ± 2778288.30	0.746

Data shown as mean ± SD

DCD, Developmental coordination disorder; TD, typical development

## Discussion

This is the first study to compare lower limb joint position sense and kinematic control of 13 to 14-year-old adolescents in a large cross-sectional sample with quantitative measures. Importantly, in agreement with previous findings, there were no differences between categorized groupings for TD and DCD adolescents. Although there was a significant correlation found between MC and kinematic control, no correlation was found between MC and knee joint position sense. A significant negative correlation was found between normalized jerk (NJ) and MC in the categories of manual dexterity, balance function, and total score of the mABC-2. Furthermore, the 80 adolescents categorized in the DCD group were found to have similar knee joint position sense and kinematic control of the lower limbs with those who are typically developed, with variability in measures observed in both groups.

In regards to joint position sense and MC, Goble et al. found that children (8–10 years) demonstrated larger position error (PE) with shorter duration and more velocity peaks compared to older adolescents (16–18 years) during an active elbow position matching task, indicating that age-related improvements in proprioceptive acuity continue throughout childhood and adolescence [26–28]. Another article stated that standard variation of position error (SDPE) of the wrist was significantly associated with the aiming & catching and balance portions of the mABC-2 score, but not with position error (PE) [29]. A large sample size study (*n* = 354) also demonstrated improvement in developmental elbow joint acuity in children aged 5–18 years for SDPE but not for PE [30]. One of the main objectives of the present study was to determine the relation between joint position sense and MC, and our results showed that neither PE nor SDPE of the knee joint correlated with any category or the total score of mABC-2. A possible reason for these results might be that the mABC-2 does not specifically target knee joint position sense or performance, but rather targets overall upper and lower limb movement. The manual dexterity and aiming & catching tasks of the mABC-2 mainly assess upper limb fine movement acuity, accuracy, speed, and coordination, whereas the balance tasks mainly focus on lower limb speed and coordination, and postural control and adjustment. Test of joint position sense, however, is not reflected in any of the components of the mABC-2 directly, and thus may not a representative picture of MC in adolescents. In addition, joint position sense reflects somatosensory input whereas MC represents sensorimotor output. A previous anatomical study found that although these neural networks overlap, they are not identical, perhaps indicating little association between proprioceptive function and MC, as reflected in the results of our study [9].

The present study also assessed the relationship between kinematic control and MC. Age has been found to be a factor in kinematic control, with better scores seen in older children, however, very little developmental studies have looked at adolescent kinematic performance [26–28]. Normalized jerk, which is the kinematic measurement of movement smoothness, was recorded during in our study. Results showed weak but significant negative correlation between NJ and MC in the categories of manual dexterity, balance function, and total mABC-2 score, indicating that a better overall MC is associated with better kinematic control in movement tasks.

Furthermore, the present study analyzed differences in joint position sense and kinematic control between adolescents with and without DCD and found no significant group differences in either category. Variability was seen within groups, with DCD adolescents exhibiting greater variability compared to TD. Tseng et al. found that in children between 9 and 11 years of age, those categorized in the DCD group performed significantly poorer in SDPE than the TD group for wrist and elbow joint position sense, but there was no group difference in PE [29, 31]. Chen et al. found significant negative correlations between proprioceptive acuity of the knee and ankle joint and balance function in children with DCD compared to TD; however, participants were spread across two age bands (7–10 years and 11–16 years) of the mABC-2 ^9^. In the present study, we proved that 13- to 14-year-old adolescents with and without DCD showed similar performance for PE and SDPE of the knee joint in a movement reproduction task. We may conclude that by the time of early adolescence, considering our findings of a relationship of kinematic control to MC, that there is a continuum between these measures with some variability rather than a distinct grouping Furthermore, as joint position sense measures small changes in movement, these differences may no longer be apparent in adolescence. Although no significant differences were found between groups in the present study, variability was higher for the DCD population (Table 5). This may indicate that the knee joint proprioceptive task may not be challenging enough to elicit differences between TD and DCD adolescents. A previous imaging study found that cortical processes seen in children with DCD are markedly different from those in TD children [32], which indicates the possible use of compensatory strategies during motor performance to mask for movement insufficiencies, especially in older children with DCD. In regard to kinematic control, our results are consistent with a previous upper limb study that showed that DCD and TD children exhibited similarities in motor performance, with group differences demonstrated only for movement smoothness (NJ). Although the present study did not conduct statistical analysis between DCD and TD groups for NJ, the coefficient of variation (CV) based on our data indicated that the DCD group showed larger CV on NJ than the TD group (CV = 8.59 and 3.79, respectively), representing that DCD adolescents had larger variation in movement smoothness [32]. Though overall performances of active movement between children with and without DCD were similar, results from previous studies indicated the possibility that children with DCD require greater engagement of motor cortical areas to control movement after initiation [32]. Differences in performance between groups may be more apparent in activities that require more complex and technical skills, such as in sports, which may have further implications on social participation, especially at a turning point in adolescent growth.

Lastly, this was a large sample size study which crossed 3 local schools in UK. According to mABC-2 norm, 80 out of 427 adolescents identified as being in the 5th percentile, and are indicated as having significant movement difficulty. Although a large number of adolescent participants were categorized as DCD with significant movement difficulty, they did not show significant group difference in either joint position sense or kinematic performance when compared with TD adolescents during the movement reproduction task (Table 5). Although studies have confirmed the validity and reliability of the mABC-2 for the younger age bands and found significant motor differences between DCD and TD children, the subjects in these studies are typically spread across a wide age band, and mainly focus on upper extremity movement [33]. There is insufficient evidence on the validity of the mABC-2 for the adolescent age band. Furthermore, the similarities in motor competence between DCD and TD children may be less apparent because adolescents are at a critical stage when internal motor processes mature rapidly and when external factors play a key role in complex motor skill development. This may also indicate that mABC-2 norms do not reflect the full picture of motor competency in adolescents. Further studies assessing the mABC-2 for adolescents are needed to expand on our findings. Finally, our sample were probable DCD which may have affect some relationships.

In summary, our study highlights on the correlation between MC, joint position sense, and kinematic control in adolescents, particularly those with DCD. We observed a stronger association between MC and kinematic control during movement tasks compared to joint position sense, suggesting that interventions focusing on improving kinematic control may significantly benefit adolescents with DCD, aiding in the development of personalized rehabilitation programs within clinical settings. Additionally, the robustness of our findings is supported by the large sample size, enhancing the generalizability of our results.

Furthermore, our findings extend beyond hospital environments, providing guidance for interventions aimed at fostering physical activity and motor skill development among adolescents in educational and community settings. However, there were limitations. Joint position sense is challenging to measure at scale and could be affected by internally predicted sensory feedback, afferent sensory feedback, or their integration [34]. The present study used an active, rather than passive, providing motor cues that aid the nervous system in predicting movement outcomes [30]. However, this method may be influenced by short-term memory and might not offer a precise measure of proprioceptive acuity [30]. Further research is needed to assess its validity and reliability in measuring lower extremity joint position sense. Additionally, incorporating peri- and postnatal history assessment in future research could provide valuable insights into early developmental factors influencing MC and movement control in adolescence. An important limitation of our study is the inclusion of “probable DCD” cases. While this categorization was necessary due to the lack of a definitive diagnosis in some participants, it may introduce variability in the data. In the present study, mABC-2 was used to assess probable DCD, which is considered as a supportive instrument for DCD diagnosis [35, 36]. Future studies should include additional diagnostic tools and steps such as DSM-5TR criteria to confirm DCD diagnosis and ensure the population corresponds to DCD more accurately.

Despite these limitations, our findings highlight the clinical relevance of considering kinematic control in interventions targeting movement difficulties in adolescents. Future studies should explore the validity and reliability of measurement methods for joint position sense in lower extremities and investigate the impact of more complex motor performance on social participation, particularly in adolescents with DCD [9].

## Conclusion

Motor competence may be associated with kinematic control during movement, rather than joint position sense. No significant correlation between knee joint position sense and MC, although there was a weak correlation between kinematic control and MC. Adolescents with DCD demonstrated identical knee joint position sense performance and kinematic control of the lower limb compared with their TD peers. Our results suggest that young people with DCD do not have difficulties with joint position sense compared with TD, and that movement control training to target kinematic control should be indicated in clinical settings and in future research on MC.

## Electronic supplementary material

Below is the link to the electronic supplementary material.

Supplementary Material 1

## Data Availability

The datasets used and/or analysed during the current study are available from the corresponding author on reasonable request.
