# Supplementary material for: Knee joint position sense and kinematic control in relation to motor competency in 13 to 14-year-old adolescents
Source: Ital J Pediatr. 2024 Sep 29;50:200. doi: 10.1186/s13052-024-01765-z (PMC11440888; doi:10.1186/s13052-024-01765-z)
Supplement: Supplementary file 1 — Supplementary Material 1 [file 13052_2024_1765_MOESM1_ESM.docx]

**Manuscript ID:** **ITJP-D-23-00459, entitled "Knee Joint Position Sense and Kinematic Control in Relation to Motor Competency in 13 to 14-Year-Old Adolescents”**

Thank you for the constructive review of our manuscript entitled “Knee Joint Position Sense and Kinematic Control in Relation to Motor Competency in 13 to 14-Year-Old Adolescents”. In the following, we have addressed all the comments on a point-by-point basis. Changes in the manuscript are underlined. We hope the revisions are satisfactory.

**Reviewers’ comments:**

**Reviewer #1:**

Dear Authors,

Thank you for addressing my comments.

I think it might be useful to elaborate a bit more on the limitiation represented by the inclusion of "probable DCD" in you study and the reasons why cases were (correctly) categorized with this phrase.

Thank you.

Best regards

**Response:**

Dear Reviewer,

Thank you for your positive feedback and for highlighting the need to elaborate on the limitation regarding the inclusion of "probable DCD" in our study. We appreciate your suggestion and have revised the manuscript to address this point. Please refer to the updated limitations section in the revised manuscript (page18 lines 9-14). Thank you.
